# A cluster of genes located in 1p36 are down-regulated in neuroblastomas with poor prognosis, but not due to CpG island methylation

**DOI:** 10.1186/1476-4598-4-10

**Published:** 2005-03-01

**Authors:** Helena Carén, Katarina Ejeskär, Susanne Fransson, Luke Hesson, Farida Latif, Rose-Marie Sjöberg, Cecilia Krona, Tommy Martinsson

**Affiliations:** 1 Department of Clinical Genetics, Institute for the Health of Women and Children, Göteborg University, Sahlgrenska Univ. Hospital-East, SE-41685 Gothenburg, Sweden; 2 Section of Medical and Molecular Genetics, Department of Paediatrics and Child Health, University of Birmingham, The Medical School, Edgbaston, Birmingham B15 2TT, UK

## Abstract

**Background:**

A common feature of neuroblastoma tumours are partial deletions of the short arm of chromosome 1 (1p-deletions). This is indicative of a neuroblastoma tumour suppressor gene being located in the region. Several groups including our have been studying candidate neuroblastoma genes in the region, but no gene/genes have yet been found that fulfil the criteria for being a neuroblastoma tumour suppressor. Since frequent mutations have not been detected, we have now analyzed the expression and promoter CpG island methylation status of the genes *UBE4B*, *KIF1B*, *PGD*, *APITD1*, *DFFA *and *PEX14 *in the 1p36.22 region in order to find an explanation for a possible down-regulation of this region.

**Results:**

The current study shows that gene transcripts in high stage neuroblastoma tumours are significantly down-regulated compared to those in low stage tumours in the 1p36.22 region. CpG island methylation does not seem to be the mechanism of down-regulation for most of the genes tested, since no methylation was detected in the fragments analyzed. One exception is the CpG island of *APITD1*. Methylation of this gene is also seen in blood from control individuals and is therefore not believed to participate in tumour development.

**Conclusion:**

The genes *UBE4B*, *KIF1B*, *PGD*, *APITD1*, *DFFA *and *PEX14 *are down-regulated in high stage NB tumours, a feature that can not be explained by CpG island methylation.

## Background

Neuroblastoma (NB) is the most common paediatric solid tumour, responsible for 15% of cancer deaths of childhood. It is a tumour of the postganglionic sympathetic nervous system that develops from immature or dedifferentiated neural crest-derived cells [1]. The distal part of chromosome 1p shows loss of heterozygosity (LOH) in 20–40% of NB tumours and has therefore been alleged to contain one or more tumour suppressor genes. We and others have previously analyzed the chromosomal region 1p36.2-3 [2-12] and we have recently focused on the gene region involving the genes: UBE4B-KIF1B-PGD-APITD1-CORT-DFFA-PEX14. These genes have been analyzed for mutations and a few have been found in rare tumours [13-17].

In search of tumour suppressor genes, the focus has in the last years moved towards epigenetics and methylation of promoter regions. Methylation of cytosines in CpG-dinucleotides is a common modification in mammalian genomes. Methylated cytosines are more susceptible to deamination, which have lead to an erosion of the number of CpG-dinucleotides. The vast majority of CpGs resides within repetitive elements and is methylated. There are also stretches of DNA rich in CpG that are gene associated, i.e. CpG islands, which are normally unmethylated [18]. Methylation is generally associated with repression of transcription. Gene regulation by methylation includes tissue-specific regulation during development and processes as X-chromosome inactivation and genomic imprinting, reviewed by Herman and co-workers. [19]. Cancer is associated with a genome-wide hypomethylation and a more gene-specific hypermethylation. Hypermethylation of CpG islands has been shown to be a common mechanism for the inactivation of tumour suppressor genes and is found in a wide range of tumour types [20-23]. According to the Knudson two-hit hypothesis, two successive mutations are required to inactivate a tumour suppressor gene and turn a normal cell into a malignant one. Inactivation could be due to deletions or mutations. Epigenetic events, such as hypermethylation of promoter-associated CpG islands have come in focus during the last decade as a route to inactivation [19].

The most common way to analyze methylation status is based on bisulphite modification of DNA [24]. In this reaction, unmethylated cytosines are deaminated to uracil, while methylated cytosines remain unconverted. The region of interest can be amplified using non-discriminating primers, amplifying both methylated and unmethylated templates in one reaction, or with methylation-specific PCR (MSP) in which methylated and unmethylated templates are amplified in separate reactions [25].

Some genes have been analyzed in NB tumours focusing on methylation status. For example, *CASP8*, on 2q33, was one of the first genes found to be methylated in neuroblastoma, with a frequency of about 40 % in primary NB tumours [26]. On 3p21.3, *RASSF1A *and *BLU *have been shown to be frequently methylated [27-29]. In a study by van Noesel and co-workers, 34 genes in 12 different chromosomal regions were analyzed in neuroblastoma cell lines [30]. A total of six genes that were methylated in at least three of the 22 cell lines were found. *CASP8*, as already known, was one of these genes, also *FLIP *at 2q33 was methylated as well as four genes in the chromosome region 8p21; *DR4*, *DR5*, *DCR1 *and *DCR2*. Genes on 1p36 were also included in this study, none of which were found to be methylated. No genes in the NB tumour suppressor candidate region on 1p36.22 were included. Alaminos and co-workers have in a study screened 45 genes in NB cell lines [31]. 34 genes were found to be methylated in at least one of the ten cell lines. The analysis also demonstrated that the percentage of methylated genes was higher in cell lines with MYCN-amplification than in those without. *UBE4B*, located in 1p36.22, was included in this study and found to be unmethylated.

The genes in the 1p36 consensus deleted region are all, except for *CORT*, associated with a CpG island in their respective promoter regions. The fact that this region is deleted in a subset of NB tumours and that frequent mutations are not found, makes it a possible candidate region for hypermethylation of genes. In the current study, we wanted to analyze the expression pattern of these genes in the 1p36.22 region using TaqMan technology and the methylation status of promoter regions associated with CpG islands, in a panel of NB cell lines and primary NB tumours.

## Results

### Expression analysis

*GUSB *was selected as an endogenous control for real-time PCR quantification, and further used as an internal reference for normalization. Real time-PCR studies of all the genes in the region on cDNA-samples showed a significant reduction of mRNA levels in high stage NB tumours compared to low stage tumour mRNA levels, ranging from 49% for *PEX14 *to 79% for *APITD1 *(Table 4) [16].

**Table 4:** Average relative gene expression (gene/GUSB).

Gene	Average relative expression in NB cell lines	Average relative expression in low stage NB	Average relative expression in high stage NB	% less expression in high stage NB compared to low stage NB
UBE4B	1.1	1.2	0.33	73%
KIF1B	0.51	3.6	0.99	72%
PGD	0.95	1.1	0.38	65%
APITD1	1.3	0.35	0.075	79%
DFFA	1.5	1.2	0.51	57%
PEX14	1.1	1.9	0.96	49%

Data analysed by TaqMan, and grouped in neuroblastoma cell lines, low stage primary tumours and high stage NB primary tumours.

**Figure 1:**
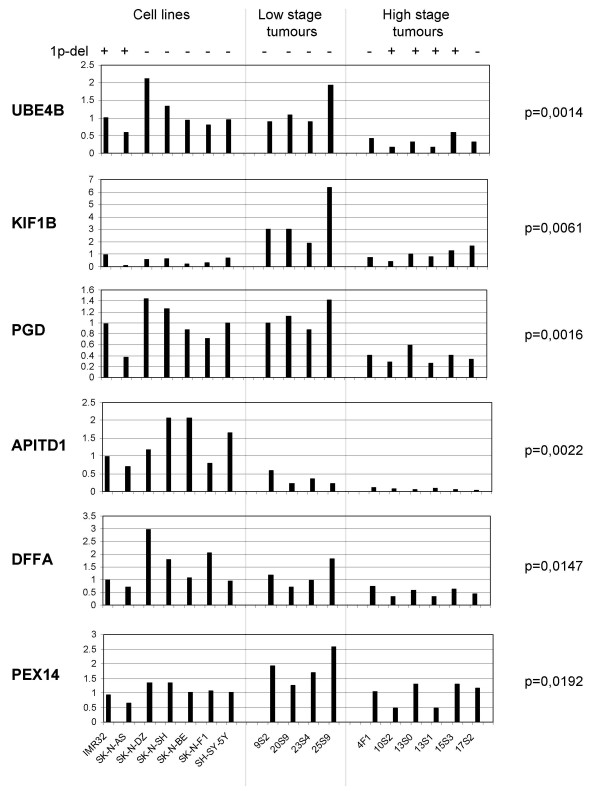
**Expression studies using the TaqMan-technique for detecting mRNA in NB cell lines and primary tumours**. The results are grouped into three groups indicated at the top; also the 1p-deletion status for each sample is indicated at the top. The genes tested are indicated to the left. The Y-axis indicates relative expression level compared to the housekeeping gene *GUSB *(gene-mRNA concentration / GUSB-mRNA concentration). The identity of each sample is indicated at the bottom.

The average expression in NB cell lines compared to primary tumours varied between the different genes in the region. Cell lines showed a higher expression of *APITD1 *and *DFFA *compared to primary tumours. Lower expression in cell lines was seen in *KIF1B *and equal levels of cell lines and stage 2 primary tumours in *UBE4B*, *PGD *and *PEX14 *(Table 4; Fig. 1). The 1p-deleted cell line SK-N-AS generally showed lower expression of the genes in the region compared to the other NB cell lines tested (Fig. 1).

### Methylation analysis

**Figure 2:**
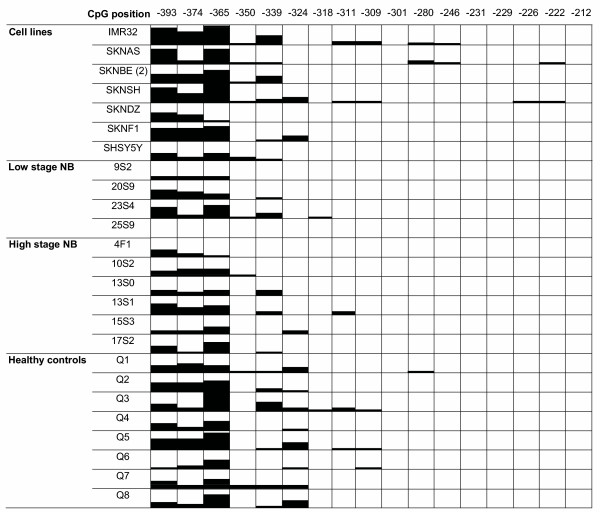
**Methylation status of APITD1**. Black boxes indicate methylation, white boxes no methylation, position relative to start codon is indicated at the top.

**Figure 3:**
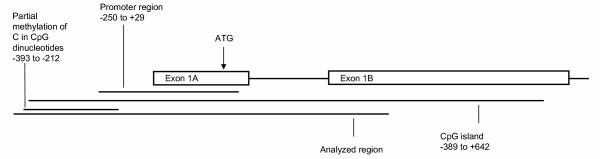
**Organization of the 5' region of APITD1**. The CpG island, predicted promoter and analyzed region including the region of partial methylation are indicated.

The analyzed fragments of the genes *UBE4B*, *KIF1B*, *PGD*, *DFFA *and *PEX14 *were not methylated in the panel of NB primary tumours and cell lines. For *APITD1*, the region -393 to -222 relative to initiation codon showed methylation. This region was therefore amplified and cloned with a T/A cloning kit in order to give a value of the percentage of clones being methylated (Fig. 2). The region contains 16 CpG sites and these sites show various degree of methylation, in different clones as well as in different samples. Methylation of cytosine in front of guanine is concentrated to the beginning of this "methylated region"; the three first cytosine bases showing the highest degree of methylated clones (Fig 3). No methylation was seen up-stream of -393 in the fragments analyzed. At some CpG sites in the region, methylation was never detected. Only one sample had no clones with methylation for any of the sites; the stage 2 tumour 20S9. Blood from control individuals also showed various degree of methylation at these sites. The cell lines did not show methylation down-stream of CpG -212 in the analyzed fragments, clones from tumours were not methylated down-stream of CpG -311. Healthy controls showed a smaller region of methylation, no methylated clones were detected down-stream of CpG -280.

## Discussion

Deletion of parts of chromosome 1p is a common feature of neuroblastoma tumours. We have previously narrowed down the shortest region of overlap of deletions (SRO) to 25 cM in our tumour material [6,7]. By including a germ cell tumour with 1p deletion we could confine the SRO to 5 cM [11]. This SRO was further confirmed when a cell line with a homozygous deletion of 500 kb was found within this region [32]. We have previously screened the 500 kb region for mutations and only rare have been detected. The aim of this study was to investigate the expression of the genes in the region and to correlate this with the methylation status. TaqMan expression data showed a clear down-regulation in gene expression of genes in the region tested when comparing high stage NB tumours (stage 3 and 4) and low stage tumours (stage 2). This down-regulation was seen regardless if the high stage tumour was 1p-deleted or not (Fig. 1). This finding further supports that this region might be involved in NB development and progression. We can speculate about some central mechanism for the expression of the genes in the entire region that is affected in tumours with poor prognosis, either due to the deletion of one allele and/or by some other mechanism. An attractive explanation for this would be methylation of CpG islands that can lead to inactivation of the respective gene. This does not seem to be the case though, since none of the genes, *APITD1 *excepted, showed methylation. Furthermore, DNA from healthy controls is also methylated in the same region of the *APITD1 *gene, making methylation a less likely explanation for the difference in *APITD1 *gene expression between advanced and favourable primary NB tumours.

CpG methylation is not the only possible explanation for the down-regulation in gene expression shown in high stage tumours. Analysis of chromatin modifications could be the next step in a further analysis of this region, in search of a mechanism that could lead to down-regulation of the genes. Deficiencies in transcription factors and upstream elements could also account for the decrease in gene transcripts. Another possibility is that the variations are due to differences in the treatment of the tumours before and after surgery. Radiation and chemotherapy could probably have a vast affect on the expression patterns of a substantial amount of genes in the tumour cell. It is however unlikely that all the genes in the analyzed region would be affected by the treatment alone, rather this might explain the differences in the level of down-regulation between different genes.

The difference in expression between NB cell lines and primary NB could be explained by the difference in cell environment between cells in culture and *in vivo*. In primary tumours, there are always some non-NB cells in the RNA preparations that might affect the gene expression results. On the other hand, genetic events happen to the cells when they get immortalized in cell culture, some genes tend to be down-regulated and others up-regulated. We can also speculate that some of these genes, for example *APITD1*, might even be so crucial that only cells with expression of this gene are capable of surviving in culture.

## Conclusion

In conclusion, in our aim to find a mechanism that could inactivate the genes in the candidate gene region we analyzed the expression status and the methylation profile of six genes. The techniques used were TaqMan real-time-RT-PCR technology and bisulphite DNA sequencing. Of the genes analyzed all were down-regulated in high staged NB tumours as compared to low stage tumours. Promoter methylation was not detected in the genes analyzed, except for the CpG island of APITD1. This methylation does not seem to be tumour specific, since methylation was also detected in healthy blood controls. Hence, the six genes *UBE4B*, *KIF1B*, *PGD*, *APITD1*, *DFFA *and *PEX14 *are down-regulated in high stage NB tumours, a feature that can not be explained by methylation, rather by a mechanism still remaining to be discovered.

## Methods

### Cell lines and patients

A panel of 10 tumours from primary NBs (4 Stage 2, all from patients with no evidence of decease at last follow-up, and 6 Stage 3 or 4 tumours, all from patients with adverse outcome) and 7 NB cell lines (IMR-32, SK-N-AS, SK-N-BE (2), SK-N-SH, SK-N-DZ, SK-N-F1 and SH-SY-5Y) were analyzed (Table 1).

**Table 1:** Clinical data for the primary tumours used in this study.

Patients/cell lines	NB stage	1p-del	Ploidi	Outcome
18F8	2A	neg		NED
20S9	2	neg		NED
23S4	2	neg	3n	NED
25S9	2	neg		NED
4F1	4	neg		DOD
10S2A	4	pos		DOD
13S0	4	pos		DOD
13S1	3	pos		DOD
15S3	4	neg/pos		DOD
17S2	4	neg		DOD

Column 3: 1p-del, 1p-deletion; neg, negative; pos, positive; neg/pos; ambiguous results based on microsatellite marker analysis (according to Martinsson et al. [6]) and FISH. Column 5: NED, no evidence of disease; DOD, dead of disease.

### Expression analysis

#### cDNA preparation

Total RNA was extracted from frozen (-70°C) NB tumour tissue using RNeasy RNA extraction kit (Qiagen, Hilden, Germany). 2.4 μg total-RNA of each sample was reversed transcribed to cDNA using Superscript II (Amersham, Buckinghamshire, UK) and random hexamer primers, all according to supplier's protocol. All cDNAs were quality tested by amplification of the housekeeping genes *UNPH *and *GAPDH*.

#### Real time PCR -Endogenous control

To select the most appropriate endogenous control for the real-time PCR quantification analysis, we tested eight different primary NB samples of different stages for their expression levels of ten commonly used housekeeping genes with TaqMan Human Endogenous Control Plate, (Applied Biosystems, Foster City, CA). Analysis was performed according to supplier's protocol. *GUSB *(β-glucuronidase) and *B2M *(β_2_-microglobulin) showed least variations in ΔC_T _levels, and were expressed at constant levels in all samples regardless of NB-stage. *GUSB *was selected, and further used as an internal reference for normalization in the real-time PCR quantification analysis (Abel et al., submitted).

#### Real time PCR- TaqMan

TaqMan primers and probes were derived from the commercially available "TaqMan^® ^Assays-on-Demand™ Gene Expression Products" (URL:). Real-time PCR was performed in 384-well plates using ABI PRISM^® ^7900HT Sequence Detection System (Applied Biosystems). Amplification reactions (10 μl) were carried out in duplicate with 0.1 μl template cDNA according to manufacturers protocol (Applied Biosystems). In each assay, a standard curve with six cDNA dilutions was recorded and two non-template controls were included.

Quantification was performed by the standard-curve method. The mean C_T_-value for duplicates were calculated, and the gene concentration (or gene copy numbers) of test samples was interpolated based on standard curves. All samples were normalized by dividing the concentration of the test gene with the concentration of the housekeeping gene β-glucuronidase (*GUSB*) in the same cDNA sample.

The logarithms of the expression levels were compared with Student's two-sided t-test on each group of tumours; low stage and high stage tumours.

### Methylation analysis

#### Bisulphite modification

DNA was phenol extracted with the use of phase lock gel (Eppendorf AG, Hamburg, Germany) according to standard procedure and was, with some minor changes, modified according to previously published papers [24,33]. Briefly, 1 μg of genomic DNA was digested with restriction endonucleases that cut close but outside the region of interest. The DNA was then denaturated in 0.3 M freshly prepared NaOH at 40°C for 15 minutes. Sodium metabisulphite (Sigma-Aldrich CO, St Louis, MO) and urea, at a final concentration of 1.73 M and 5.36 M respectively, were added in order to sulphonate the unmethylated cytosines, along with hydroquinone (0.5 mM). Conversion was carried out at 55°C for 16 hours, with a temperature rise to 95°C for 30 seconds every third hour. DNA was purified with Wizard DNA clean up system (Promega Corporation, Madison, WI) according to the manufactures instructions and desulphonated in 0.3 M NaOH at 37°C for 15 minutes. DNA was then precipitated in ethanol, resuspended in distilled H_2_O and stored at -20°C.

#### Promoter analysis and DNA amplification

The putative promoter regions of the genes were predicted using Genomatix Promoter Inspector software (URL: ) and CpG islands with MethPrimer software (URL: ; Table 2) [34]. These regions, or parts of them, were amplified with one primer pair, or if needed, with semi-nested primers (Table 3). The methylation status was analyzed using bisulphite sequencing. Conditions for PCR amplification were 1× PCR Gold Buffer (Applied Biosystems), 0.5 mM dNTPs, 2.0–3.0 mM MgCl_2_, 0.4 μM of forward and reverse primers respectively and 1 unit of AmpliTaq Gold, in a total volume of 50 μl. Reactions were denatured at 95°C for 10 min followed by 5 cycles of 95°C for 1 min, 49–55°C for 2 min, 72°C for 3 min and 30 cycles of 95°C for 30 sec, 49–55°C for 2 min, 72°C for 1 min 30 sec and ending with 10 min extension at 72°C. The PCR products were immediately sequenced or cloned into a sequencing vector using the TOPO T/A cloning kit (Life Technologies Invitrogen, Carlsbad, CA), where after 10–30 clones were picked. PCR products were purified with ExoSAP-IT™ (USB Corporation, Cleveland, Ohio) and sequencing was carried out using forward or reverse primer with ABI Prism BigDye™ cycle sequencing Ready Reaction Kit (Applied Biosystems). The samples were analyzed in an ABI 3100 or an ABI 3730 Genetic Analyzer.

**Table 2:** Putative promoter regions and CpG island predictions.

Gene	Promoter region	CpG island	Amplified region
UBE4B	-1212 to -492	-1157 to -150	-959 to -494
KIF1B	-22392 to -21141	-22444 to -20935	-22517 to -21874
PGD	-185 to +7	-680 to +728	-157 to +144
APITD1^a^	-250 to +30	-389 to +643	-418 to +321
DFFA	+65 to +264	-164 to +256	-266 to +142
PEX14	-	-2806 to -2362	-2752 to -2481

^a ^For *APITD1*, the methylation status was analyzed for three different fragments. Column 2: Putative promoter regions according to Genomatix Promoter Inspector software. No promoter region was detected in *PEX14*. Column 3: CpG island prediction by MethPrimer (for CpG island criteria, see materials and methods). Column 4: Region amplified with the primers used in this study.

**Table 3:** PCR primers for amplification; all primers are designed for the sense strand.

Gene	Primer	Sequence	Length of fragment (bp)	Accession number
*UBE4B*	FP	5'-TTGTTAGTTTATTTGGTTTAGGTT-3'	466	NM_006048
	RP	5'-TAACAAAACCCAACACTATAAAAAAAACCCCT-3'		
*KIF*	FP	5'-TTTTTAAGGGTATTTTTTAGAAGGG-3'	644	NM_015074
	RP	5'-ACTATAACCAATCACAACACAAAACTC-3'		
*PGD*	FP-A	5'-GTGAGTTGTTATGGTTATAGTTG-3'	301	NM_002631
	FP-B	5'-ATGGTGTGGTTTTATGGTTTTATTT-3'		
	RP	5'-CAAAATCACAAAACCCCAAATAA-3'		
*APITD1*	1FP-A	5'-GATTTTGTAAGATATATTTGAGGTAT-3'	231	chr1_29_927.b
	1FP-B	5'-ATGGAGTTTTTGATAATGTGTATTG-3'		
	1RP	5'-AACCCCCTACTCAACTTACTCTAC-3'		
	2FP-A	5'-ATTAGGTTTTGGGGTGTAGTAGTGAT-3'	199	
	2FP-B	5'-GTAGAGTAAGTTGAGTAGGGGGTTG-3'		
	2RP	5'-ACCCTAAACAAAAACAAAAAAAC-3'		
	3FP-A	5'-GTAGAGTAAGTTGAGTAGGGGGTTG-3'	350	
	3FP-B	5'-TTGTTTTTGTTTAGGGTCGGTT-3'		
	3RP	5'-CAAAACCAAAAAATAACCTCTC-3'		
*DFFA*	FP	5'-AAGTTAAAAATAATTTTTAGGTTGAAT-3'	407	NM_004401
	RP	5'-ACCAACCCTTACTCCTCAAATCT-3'		
*PEX14*	FP	5'-TGATTAGTTAGGTTTTAGAAAGATGG-3'	333	NM_004565
	RP	5'-CAAATAAAACCAAAAATACTAACAAAC-3'		

Column 2: FP, forward primer; RP, reverse primer; FP-A and FP-B, primers used for semi-nested PCR together with the same reverse primer. Column 5: UCSC Genome Browser August 2001 draft sequence was used as reference sequence (URL: ).

## Authors' contributions

HC participated in the design of the study, carried out the methylation analysis and drafted the manuscript. SF, LH and FL participated in parts of the methylation study. KE and CK carried out the TaqMan runs. KE also contributed to drafting the manuscript. RMS participated in the cloning. TM coordinated the study. All authors read and approved the final manuscript.
